# The Rescue and Characterization of Recombinant, Microcephaly-Associated Zika Viruses as Single-Round Infectious Particles

**DOI:** 10.3390/v11111005

**Published:** 2019-10-31

**Authors:** Chien-Yi Lu, Chen-Sheng Lin, Hsueh-Chou Lai, Ya-Wen Yu, Chih-Yi Liao, Wen-Chi Su, Bo-Han Ko, Young-Sheng Chang, Su-Hua Huang, Cheng-Wen Lin

**Affiliations:** 1 Department of Medical Laboratory Science and Biotechnology, China Medical University, Taichung 404, Taiwan; cylu0424@gmail.com (C.-Y.L.); chloe830628@gmail.com (Y.-W.Y.); angelliaoj302@gmail.com (C.-Y.L.); pig60666@gmail.com (B.-H.K.); a0989016192@gmail.com (Y.-S.C.); 2 Division of Gastroenterology, Kuang Tien General Hospital, Taichung 433, Taiwan; b8401126@yahoo.com.tw; 3 School of Chinese Medicine, China Medical University, Taichung 404, Taiwan; t674233@ms54.hinet.net; 4 Division of Hepato-gastroenterology, department of internal medicine, China Medical University Hospital, Taichung 404, Taiwan; 5 Graduate Institute of Biomedical Sciences, China Medical University, Taichung 404, Taiwan; t23514@mail.cmuh.org.tw; 6 Department of Biotechnology, Asia University, Wufeng, Taichung 413, Taiwan; shhuang@asia.edu.tw; 7 Chinese Medicine Research center, China Medical University, Taichung 404, Taiwan

**Keywords:** Zika virus, single-round infectious particle, replicon, reporter, cell susceptibility

## Abstract

Zika virus (ZIKV) is transmitted by *Aedes* mosquitoes and exhibits genetic variation with African and Asian lineages. ZIKV Natal RGN strain, an Asian-lineage virus, has been identified in brain tissues from fetal autopsy cases with microcephaly and is suggested to be a neurotropic variant. However, ZIKV Natal RGN strain has not been isolated; its biological features are not yet illustrated. This study rescued and characterized recombinant, single-round infectious particles (SRIPs) of the ZIKV Natal RGN strain using reverse genetic and synthetic biology techniques. The DNA-launched replicon of ZIKV Natal RGN was constructed and contains the EGFP reporter, lacks *prM-E* genes, and replicates under CMV promoter control. The peak in the ZIKV Natal RGN SRIP titer reached 6.25 × 10^6^ TCID50/mL in the supernatant of prM-E-expressing packaging cells 72 h post-transfection with a ZIKV Natal RGN replicon. The infectivity of ZIKV Natal RGN SRIPs has been demonstrated to correlate with the green florescence intensity of the EGFP reporter, the SRIP-induced cytopathic effect, and ZIKV’s non-structural protein expression. Moreover, ZIKV Natal RGN SRIPs effectively self-replicated in rhabdomyosarcoma/muscle, glioblastoma/astrocytoma, and retinal pigmented epithelial cells, displaying unique cell susceptibility with differential attachment activity. Therefore, the recombinant ZIKV Natal RGN strain was rescued as SRIPs that could be used to elucidate the biological features of a neurotropic strain regarding cell tropism and pathogenic components, apply for antiviral agent screening, and develop vaccine candidates.

## 1. Introduction

Zika virus (ZIKV) was first isolated in monkeys in Uganda in 1947, and then detected in *Aedes africanus* mosquitoes in 1948. ZIKV belongs to a mosquito-borne flavivirus of the family Flavivirus, which spread from Africa to south-eastern Asia through transmission by *Aedes* mosquitoes, such as *A. albopictus, A. Aegypti,* and *A. Polynesiensis*. ZIKV exhibits genetic variation that is classified into African and Asian lineages [1]. ZIKV infection causes self-limiting, mild illnesses, such as low-grade fever, headaches, rash, myalgia, arthralgia, and conjunctivitis; fewer than 20 human ZIKV cases were reported from the 1960s to 1980s [2]. Recently, the ZIKV Asian-lineage strains spread from south-east Asia toward the Pacific, leading to three large outbreaks on Yap island (Micronesia) in 2007, French Polynesia in 2013–2014, in Brazil in 2015–2016, and then across the Americas in 2016 [3]. The epidemic ZIKV Asian strains evolved into highly virulent and neurotropic variants, resulting in a dramatic increase in cases of congenital, ZIKV-associated microcephaly in newborns and Guillain–Barre syndrome (GBS) in adults during these three outbreaks [3]. The epidemic ZIKV Asian strains have also been transmitted via vertical, sexual, and blood transfusion routes [4]. Therefore, the WHO Emergency Committee announced that ZIKV is considered a “Public Health Emergency of International Concern” (PHEIC) in November 2016 [5]. Nowadays, the epidemic ZIKV Asian strains have broadened worldwide; laboratory-confirmed symptomatic ZIKV cases are still reported [6]. ZIKV’s emergence has become an extraordinary and enduring challenge to public health; thus, there is a great need to understand the features of the epidemic ZIKV Asian strains.

ZIKV, like other flaviviruses, such as Dengue virus, Japanese encephalitis virus (JEV), and West Nile virus, possesses a single-strand, positive-sense RNA genome in a small, enveloped virion [7]. The flavivirus genome, nearly 11 kb in length, encodes a large open reading frame (ORF) flanked by two noncoding regions at the 5’ and 3’-ends, which translates into a large polyprotein that is divided into three structural proteins (capsid (C), membrane (prM/M), and envelope (E)) and seven non-structural proteins (NS1, NS2A, NS2B, NS3, NS4A, NS4B, and NS5) through the proteolytic process of the viral proteases (NS2B–NS3) and host cell proteases. E protein is the surface protein for receptor binding and cell membrane fusion. NS3 is a multidomain protein with an N-terminal protease (NS3pro), C-terminal RNA triphosphatase (NS3 RTPase), and helicase (NS3hel). NS5 consists of an N-terminal methyltransferase (NS5 MTase) and C-terminal RNA polymerase (NS5RdRp). Phylogenetic analysis of ZIKV African and Asian-lineage strains isolated from 1947 to 2017 indicates that some mutations accumulated in the epidemic ZIKV Asian strains in 2015–2016 [8,9]. Epidemic ZIKV Asian strains contain 75 amino acid substitutions compared with their African lineage strains, five of which are located in C, nine in prM, 10 in E, four in NS1, five in NS2A, two in NS2B, nine in NS3, one in NS4A, nine in NS4B, and 21 in NS5 [8,9]. The serine-to-asparagine substitution (Ser^139^→Asn^139^ (S139N0) in prM increases ZIKV infectivity in neural progenitor cells and the severity of fetal microcephaly in a mouse model [10]. The alanine-to-valine substitution (A^984^→V^984^ [A984V]) in NS1 induces the inhibition of interferon-β production and leads to viral immune escape [11]. In addition, comparison between pre-epidemic and epidemic ZIKV Asian strains indicated 24 amino acid substitutions within prM (2), E (3), NS2A (1), NS3 (3), NS4B (7), and NS5 (8), such as T773M in E, Y2082H in NS3, L2451S in NS4B, and T2630V in NS5 [8,9]. The mutations in epidemic ZIKV Asian strains in comparison with pre-epidemic Asian and African strains might be determinants for their unique features; considering the increase in virulence and persistent infection, these mutations are worth understandingly thoroughly.

Reverse genetics is an effective tool for preparing replicons and infectious clones of flaviviruses, in order to study their mechanisms of viral replication and pathogenesis and to identify potential antiviral compounds [12,13]. The full-length, infectious cDNA clones of flaviviruses under cytomegalovirus’s (CMV’s) promoter-driven transcription have been widely used to generate infectious particles in vitro. However, full-length flavivirus cDNA clones are highly toxic to *Escherichia coli*, which causes instability of flavivirus cDNAs and increases the difficulty of constructing flavivirus cDNA clones [14]. DNA-launched replicons of dengue virus, West Nile virus, and JEV contain the 5’ and 3’-noncoding regions, partial C gene, and the complete gene fragment of all non-structural proteins under the control of the CMV promoter [12]. CMV promoter-driven viral RNA subgenomes enable self-replication in transfected cells with DNA-launched replicons, but they possess no infectious particle production due to lacking the coding region of structural proteins. The approach using two plasmids with the structural genes (C, prM, and E) and the remaining genome as the replicon has been established to produce single-round infectious particles (SRIPs). The packaging cells carrying the recombinant plasmid encoding the viral structural proteins are transfected with DNA-launched replicons and then self-replicate viral subgenomes and express all viral proteins, allowing viral assembly and release as SRIPs. Several flavivirus, replicon-based SRIPs, including JEV, dengue virus, West Nile virus, and tick-borne encephalitis virus have been generated and demonstrated as safer vaccine candidates [15,16,17]. Subsequently, replicon-based SRIPs are suitable systems for investigating the mechanism of the ZIKV life cycle, ZIKV-host interaction, and virulence.

The ZIKV Natal RGN strain that is associated with microcephaly was recognized as the representative, epidemic, ZIKV Asian strain in 2015–2016 [18]. The ZIKV Natal RGN strain is detected in brain tissues from fetal autopsy cases with microcephaly in the Natal region of Brazil; its genome has been sequenced using next-generation sequencing (GenBank accession number KU527068) [15]. The ZIKV Natal RGN strain contains a unique genotype and phenotype; therefore, this study aimed to generate SRIPs of the ZIKV Natal RGN strain using synthetic and reverse genetic technologies. This paper characterizes the infectivity of ZIKV Natal RGN SRIPs in different cell types. Moreover, the paper analyzes the expression pattern of type I interferons and apoptosis-related genes induced by ZIKV Natal RGN SRIPs. 

## 2. Material and Methods

### 2.1. Cells

TE671 (human, rhabdomyosarcoma/muscle), SF268 (human brain, glioblastoma/astrocytoma), and ARPE-19 (human, retinal pigmented epithelium) cells were cultured in minimum essential medium containing 10% fetal bovine serum, 2 mM glutamine, 1 mM pyruvate, and 1× penicillin-streptomycin at 37 °C with 5% CO_2_.

### 2.2. Generation of the ZIKV Natal RGN Replicon

**Figure 1:**
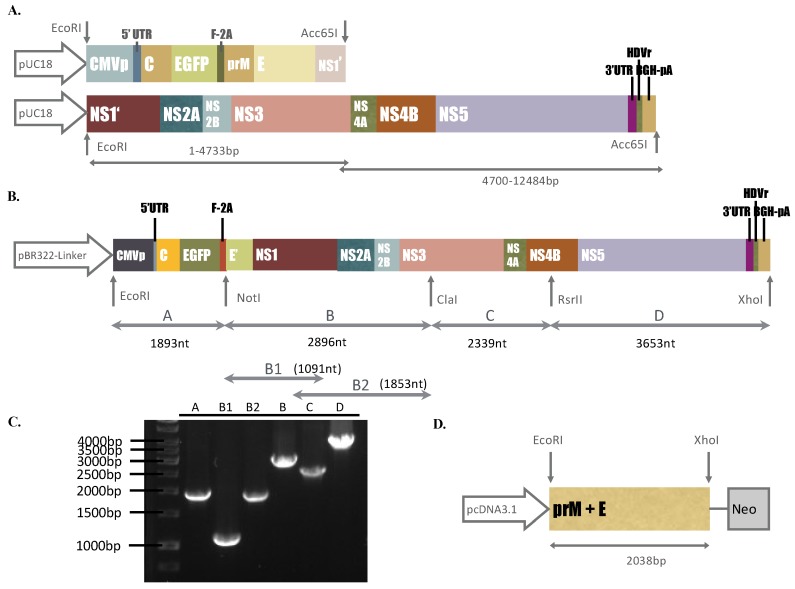
Construction of the pBR322-based ZIKV Natal RGN replicon and pcDNA3.1-ZIKV prME. Two synthetic DNA segments in the pUC18 plasmid contained the entire ZIKV Natal RGN strain genome: CMVp, EGFP, FMDV-2A, and BGH-pA sequences (**A**). Four PCR products (Fragments A–D) were cloned into the indicated restriction sites (EcoRI, NotI, ClaI, RsrII, and XhoI) of the pBR322 plasmid and then assembled as the in-frame fusion of the ZIKV Natal RGN replicon with the EGFP reporter under CMV-promoter control (**B**). Those four PCR products were analyzed using agarose gel electrophoresis (**C**). The PCR product of ZKIV *prM* and *E* genes was cloned into restriction sites (EcoRI and XhoI) of the pcDNA3.1-His-C plasmid (**D**).

Components of two DNA segments we synthesized, I and II, consisted of the CMV immediate-early promoter (CMVp), cDNA fragments of the ZIKV Natal RGN genome (GenBank accession number KU527068)， enhanced green fluorescent protein (EGFP), FMDV-2A (F-2A), hepatitis delta virus ribozyme (HDVr), and bovine growth hormone polyA signal (BGH-pA), which were purchased from Bio Basic Inc. *(*Ontario, Canada). The construction of synthesized DNA fragments, plasmid information, and restriction enzyme digestion analysis of the DNA fragments we purchased are shown in Supplementary Figures S1 and S2 and Figure 1A. To construct the ZIKV Natal RGN replicon, the plasmid pBR322-Linker, described in our prior report [15], was chosen as the vector to assemble fragments A, B, C, and D of the CMVp-driven ZIKV Natal RGN replicon with the EGFP reporter via the cloning sites of EcoRI, NotI, ClaI, RsrII, and XhoI (Figure 1B). Fragments A, C, and D were generated using the Platinum^®^ PCR Supermix High Fidelity reaction with the DNA segments we synthesized, I and II, as the templates (Life Technology, Carlsbad, CA, USA). Fragment B was produced through two-round PCR with the Gibson Assembly reaction of two first-round PCR products (B1 and B2) as the template (New England Biolabs, Ipswich, MA, USA). The pairs containing the restriction enzyme site(s) indicated, are listed in Supplementary Table S1. The resultant plasmid carrying the CMVp-driven ZIKV Natal RGN replicon with the EGFP reporter was propagated in *E. coli* DH5α and then sequenced by Sanger sequencing assays with sequencing primers (Supplementary Table S2). The nucleotide and deduced amino acid sequence alignment analysis of the CMVp-driven ZIKV Natal RGN replicon and its parent strain (GenBank accession number KU527068) was performed using the Lasergene DNASTAR Megalign software.

### 2.3. Functional Analysis of the ZIKV Natal RGN Replicon Using RT- PCR and Immunofluorescent Staining

To detect self-amplifying RNA genomes of the CMVp-driven ZIKV Natal RGN replicon, the synthesis of positive and negative-sense RNA subgenomes in vitro was examined using SYBR Green-based real-time PCR. Total RNAs of TE-671 cells transfected with the ZIKV Natal RGN replicon were extracted using the PureLink Mini Total RNA Purification Kit (Thermo Fisher Scientific, Waltham, MA, USA), reverse transcribed into cDNA with specific-capture primers, and measured using real-time PCR with ZIKV *NS5*-specific primer pairs (Supplementary Table S3), according to our prior report [19]. Relative levels of self-amplifying ZIKV sense and antisense genomes were normalized to glyceraldehyde 3-phosphate dehydrogenase (GAPDH). The absolute copy number of the ZIKV genome was determined according to the standard curve of real-time PCR for serial dilutions of the plasmid containing the ZIKV *NS5* gene at a known concentration. To explore the expression of replicon-based EGFP and ZIKV reporter proteins, replicon-transfected cells were initially photographed using immunofluorescence microscopy; then, an immunofluorescence assay (IFA) was performed with primary antibodies against ZIKV NS1 and NS5 (GeneTex, Inc., Taiwan) and secondary AF546 goat anti-rabbit IgG (Thermo Fisher Scientific). The immunofluorescent staining assay was carried out as described in our prior report [15]. The fluorescence intensity of replicon-based EGFP and ZIKV proteins in ZIKV Natal RGN replicon-transfected cells was counted by Image J.

### 2.4. Establishment of a Packaging Cell Line Expressing ZIKV prM and E Proteins

To establish the packaging cells expressing ZIKV structural proteins, the *prM/M-E* gene was amplified using PCR with specific primers (Supplementary Table S1) and synthesized DNA segment I as the template. The PCR product of the ZIKV *prM-E* gene was digested with EcoRI and XhoI, and then cloned into the EcoRI and XhoI sites of the expression plasmid pcDNA3.1-HisC. The resultant plasmid pcDNA-ZIKV prME was transfected with TE-671 cells at 90% confluence in a 6-well plate with Lipofectamine LTX (Invitrogen, Carlsbad, CA, USA) according to the manufacturer’s guidelines. The transfected cells were selected in the culture media with 500 µg/mL G418 for 2 weeks; the expression of ZIKV prM and E proteins in a stably transfected cell line (packaging cell line) was validated by real-time RT-PCR with ZIKV *E*-specific primers (Supplementary Table S3) and immunofluorescence staining with primary antibodies against ZIKV E protein (GeneTex, Inc.), as described above.

### 2.5. Production of ZIKV Natal RGN SRIPs

The packaging cells grown at 90% confluence in 6-well plates were transfected with or without the ZIKV Natal RGN replicon using Lipofectamine LTX. The cytopathic effect (CPE), replicon-based EGFP and ZIKV proteins, and viral sense and antisense genomes in mock and transfected cells, were measured 24 and 72 h post-transfection using immunofluorescent staining assays, as described above. Moreover, real-time RT-PCR assays with *NS5*-specific primer pairs, listed in Supplementary Table S3, were performed to examine the positive and negative-sense viral genomes in replicon-transfected packaging cells, as well as the viral genome in SRIPs. ZIKV Natal RGN SRIPs in the cultured media of transfected packaging cells were concentrated using the PEG Virus Precipitation Kit (Abcam, Cambridge, MA, USA). The cultured media were centrifuged at 3200× *g* for 15 min at 4 °C; then, the supernatant (10 mL) was collected from each and incubated with 2.5 mL PEG solution overnight at 4 °C. The pellets of ZIKV Natal RGN SRIPs were harvested after centrifugation at 3200× *g* for 30 min at 4 °C, re-suspended in 100 μL Virus Re-suspension Solution, and then stored at −80 °C.

### 2.6. Antigenicity, Titer, and Infectivity of ZIKV Natal RGN SRIPs

To analyze the antigenicity of ZIKV Natal RGN SRIPs, 10 μL each of one-fold and 10-fold SRIP stock dilution was spotted onto a nitrocellulose membrane for the dot-blot assay with anti-ZIKV E antibodies. The membrane was subsequently blocked with 5% skim milk in TBST (Tris-Buffered Saline, 0.1% Tween-20) buffer at 4 °C for 2 h, incubated overnight with anti-ZKIV E antibodies (GeneTex, Inc., Taiwan), and then reacted with HRP-conjugated anti-mouse IgG antibodies (Invitrogen, Carlsbad, CA, USA) after washing with TBST. Immunoreactive signals for the E proteins of ZIKV Natal RGN SRIPs were amplified with ECLTM Western Blotting Detection Reagents (GE Healthcare, Chicago, IL, USA), and then imaged by the Multi-function Gel Image System (MultiGel-21) (Gentaur, San Jose, CA, USA). The infectious titer of the ZIKV Natal RGN SRIP stock was determined by a median tissue culture infectious dose (TCID50) assay. Serial dilutions of the ZIKV Natal RGN SRIP stock were added and incubated on the 90% confluent monolayer of packaging cells in 96-well plates. After incubating for 72 h at 37 °C, the CPE in each well was observed and recorded to determine the TCID50 titer of the ZIKV Natal RGN SRIP stock. In the infectivity assay, packaging cells were cultured in 6-well plates overnight and infected with different doses of ZIKV Natal RGN SRIPs at multiplicity of infections (MOIs) of 3, 0.3, 0.03, and 0.003. CPE, replicon-driven EGFP, and ZIKV NS1 expression in the SRIP-infected cells were examined 72 h post-infection, and fluorescence microscopy and immunofluorescent staining were performed as described above. 

### 2.7. Cell Line Susceptibility and Attachment to ZIKV Natal RGN SRIPs 

In cell line susceptibility assays, TE-671, SF-268, and ARPE-19 cells were infected with ZIKV Natal RGN SRIPs at a MOI of 0.15 TCID50/cell. After incubating for 72 h, the cells were photographed to examine CPE and explore replicon-driven EGFP reporter expression using immunofluorescence microscopy. Moreover, the cells were fixed with paraformaldehyde at 4 °C for 1 h, and then immunofluorescence staining was performed with anti-ZIKV NS1 antibodies, as mentioned above. In the attachment assay, ZIKV Natal RGN SRIPs at a dose of 10^5^ TCID50/well (MOI = 0.5 TCID50/cell) were added onto the monolayers of ARPE-19, TE671, and SF-268 cells and incubated at 4 °C for 1 h. After washing with cold PBS, ZIKV Natal RGN SRIPs attached on the cell surface were harvested, and the relative levels of extracted viral genomes were quantitated using two-step, SYBR, Green I, real-time RT-PCR with ZIKV *NS5*-specific primer pairs, as described above. 

### 2.8. Statistical Analysis

All data from 3 independent experiments were analyzed using Student’s *t*-tests or χ^2^ tests. Statistical significance for each assay was judged at *p* < 0.05.

## 3. Results

### 3.1. Construction of the DNA-Launched ZIKV Reporter Replicon 

The ZIKV Asian Natal RGN prototype strain, a microcephaly-associated ZIKV, was not isolated from the brain tissues from fetal autopsy cases; therefore, this study intended to recover the recombinant ZIKV Natal RGN strain using reverse genetics and synthetic biology techniques based on the published genome sequence (GenBank accession number KU527068). Two synthetic DNA segments in the pUC18 plasmids comprised the entire ZIKV Natal RGN strain genome， CMVp, EGFP, FMDV-2A, and BGH-pA sequences (Supplementary Figures S1 and S2 and Figure 1A,B). The synthetic DNA segments were used as templates via PCR to amplify the gene fragments (A–D) that were cloned into the low-copy-number plasmid pBR322 to construct a DNA-launched ZIKV Natal RGN replicon under the control of the CMV promoter (Figure 1B,C). Fragment A consisted of CMVp, ZIKV 5’-UTR, ZIKV C protein, the reporter EGFP, and FMDV-2A. Fragment B containing the 73-residue C-terminal region of ZIKV E protein, NS1, NS2A, NS2B, and the N-terminal region of NS3, was produced using Gibson Assembly cloning (Figure 1C). In order to join fragments B and C, the reverse primer for fragment B and the forward primer for fragment C contained the restriction enzyme site ClaI (Supplementary Table S1), which is recognized as the marker _nt5156_ATCGAT_5161_ of the replicon in which thymidine at nucleotide 5156 of the ZIKV Natal RGN genome was exchanged with adenosine, but it caused a silent mutation (Figure 1B). Interestingly, the RsrII restriction site within the NS4B gene *(_nt7473_*CGGACCG_7479_) of the ZIKV Natal RGN genome was a unique restriction site for ligation between fragments C and D. In addition, HDVr was fused with the 3*’-*UTR *of* ZIKV in the replicon to verify the accuracy of the 3’-end of the transcribed ZIKV Natal RGN RNA genomes (Figure 1B,C). Furthermore, sequencing analysis of the ZIKV Natal RGN replicon indicated that only two amino acid substitutions appeared within the NS5 protein (L3277R and E3280N) (Supplementary Figure S3). Therefore, the DNA-launched ZIKV Natal RGN replicon was effectively constructed to generate the in-frame fusion of ZIKV C protein, reporter EGFP, FMDV-2A, the 73-residue C-terminal region of ZIKV E protein, and all non-structural ZIKV proteins under the control of the CMV promoter.

### 3.2. Translation and Transcription of the DNA-Launched ZIKV Natal RGN *Replicon*

**Figure 2:**
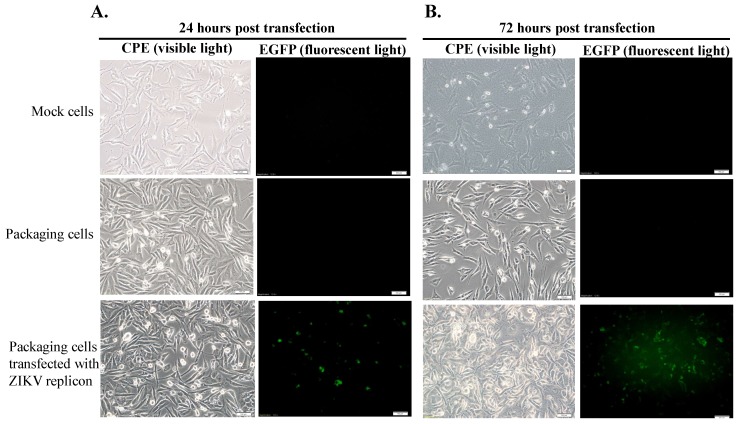
Analysis of the cytopathic effect (CPE) and EGFP reporter expression in prM-E-expressing packaging cells transfected with the ZIKV Natal RGN replicon. CPE and EGFP reporter expressions in mock cells (top), packaging cells (middle), and replicon-transfected packaging cells (bottom) were photographed using light and fluorescence microscopes 24 (**A**) and 72 (**B**) h post-incubation. Scale bar, 100 μm.

**Figure 3:**
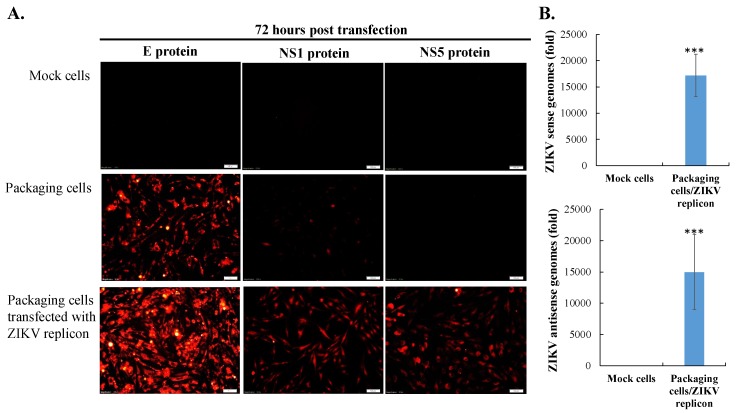
Analysis of viral proteins and genome synthesis in packaging cells transfected with the ZIKV Natal RGN replicon. ZIKV E, NS1, and NS5 expressions in mock cells (top), packaging cells (middle), and replicon-transfected packaging cells (bottom) were examined using the indicated primary antibodies and Alexa Fluor 546-conjugated secondary antibodies (**A**). Finally, cell imaging was conducted via immunofluorescence microscopy. In addition, relative copy numbers of sense (top) and antisense (bottom) genomes in the indicated cells were quantitated 72 h post-transfection using real-time PCR, and were then normalized to GAPDH mRNA (**B**). ***, *p* value < 0.001 compared with mock cells. Scale bar, 100 μm.

Initially, TE671 cells were transfected with pcDNA3.1-ZIKV prM-E, selected with G418 for 2 weeks, analyzed to examine ZIKV prM and E protein expression, and then recognized as the packaging cell line (Figure 1D, Figure 2A middle, Figure 3A middle). Subsequently, the packaging cells were transfected with the ZIKV Natal RGN replicon, and then CPE and the EGFP reporter expressions were examined (Figure 2). In the packaging cells, EGFP reporter expression and CPE showed slight levels 24 h post-transfection, but exhibited significant changes 72 h after transfection with the ZIKV Natal RGN replicon (Figure 2 bottom). Moreover, in vitro translation and transcription of the ZIKV Natal RGN replicon in packaging cells were further characterized (Figure 3). Immunofluorescent staining indicated that the ZIKV E C-terminus, NS1, and NS5 proteins were massively expressed in replicon-transfected packaging cells (Figure 3A bottom), but not in mock and packaging cells (Figure 3A top and middle). The real-time, reverse transcription PCR assay showed large amounts of positive and negative-sense ZIKV subgenomes in replicon-transfected packaging cells 72 h post-transfection， but not in mock cells (Figure 3B top and bottom). The results demonstrated efficient translation and replication of the ZIKV subgenomes in the packaging cells using the DNA-launched ZIKV Natal RGN replicon.

### 3.3. Production and Infectivity of ZIKV Natal RGN SRIPs

**Figure 4:**
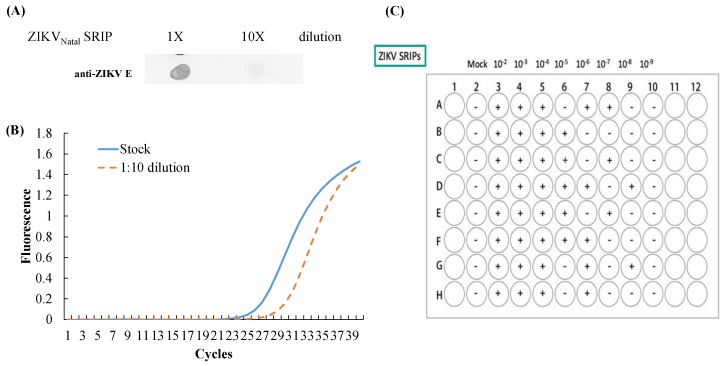
Antigenicity and quantitative analysis of ZIKV Natal RGN single-round infectious particles (SRIPs). The antigenicity, relative viral genome levels, and titer of SRIPs collected from the supernatant of the packaging cells transfected with the ZIKV Natal RGN replicon were analyzed using dot blot (**A**), real-time RT-PCR (**B**), and TCID50 (**C**) assays, respectively.

To examine the production of ZIKV Natal RGN *SRIPs*, the supernatant of replicon-transfected packaging cells was harvested 72 h post-transfection and analyzed using dot-blotting, real-time RT-PCR, and TCID50 assays (Figure 4). The dot-blotting assay with anti-ZIKV E protein demonstrated the antigenic structure of the E protein (Figure 4A), and real-time RT-PCR assay elucidated the viral subgenomes of ZIKV Natal RGN SRIPs from the supernatant of the replicon-transfected packaging cells (Figure 4B). Moreover, the titer of ZIKV Natal RGN SRIPs in the supernatant was determined by TCID50 assay with the packaging cells. The titer of the ZIKV Natal RGN SRIP stock was 6.25 × 10^6^ TCID50/mL (Figure 4C). 

**Figure 5:**
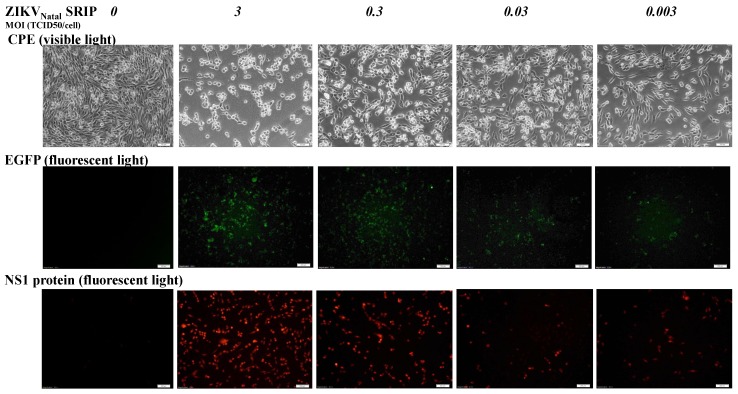
Infectivity of ZIKV Natal RGN SRIPs in prM-E-expressing packaging cells. The cells were infected with ZIKV Natal RGN SRIPs at MOIs of 3, 0.3, 0.03, and 0.003. The cytopathic effect (top) and the EGFP reporter (middle) in SRIP-infected cells were photographed using light and fluorescence microscopy 72 h post-infection. In addition, infected cells were washed, fixed, and stained by anti-NS1 antibodies and Alexa Fluor 546-conjugated secondary antibodies (bottom). Finally, cell imaging was taken by immunofluorescence microscopy. Scale bars, 100 μm for CPE and EGFP images (top and middle) and 200 μm for the NS1 image.

To examine the infectivity of ZIKV Natal RGN SRIPs in vitro, the packaging cells were infected with 10-fold dilutions (MOIs of 3, 0.3, 0.03, and 0.003) of ZIKV Natal RGN SRIPs to determine SRIP-induced CPE and SRIP-driven expression of EGFP reporter and ZIKV NS1 (Figure 5). ZIKV Natal RGN SRIPs dose-dependently induced CPE and expressed SRIP-driven EGFP in packaging cells 72 h post-infection (Figure 5 top and middle). In addition, immunofluorescent staining revealed dose-dependent expression of ZIKV NS1 protein in ZIKV Natal RGN SRIP-infected packaging cells (Figure 5 bottom). 

### 3.4. Cell Susceptibility to ZIKV Natal RGN SRIPs

**Figure 6:**
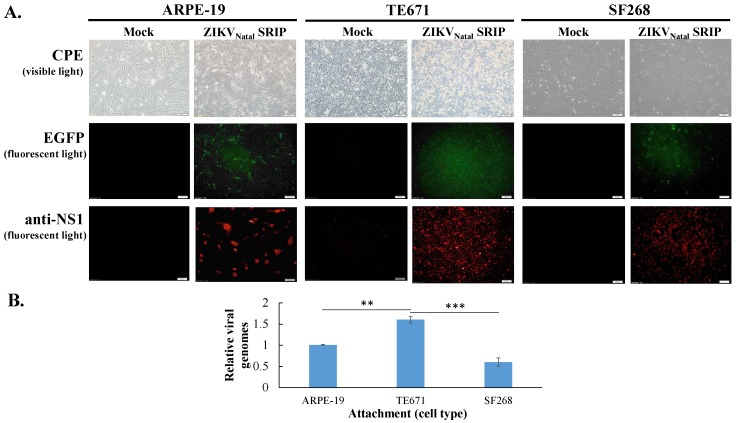
Cell susceptibility to ZIKV Natal RGN SRIPs. Three cell lines, ARPE-19, TE671, and SF268, were infected with SRIPs at an MOI of 0.15 TCID50/cell. The cytopathic effect (top), EGFP reporter (middle), and ZIKV NS1 protein (bottom) in SRIP-infected cells were photographed using light and fluorescence microscopy 72 h post-infection (**A**). In addition, relative viral genome levels from SRIPs attached to the surfaces of these cell lines were measured using real-time RT-PCR mRNA (**B**). **, *p* value < 0.01; ***, *p* value < 0.001 compared with mock cells. Scale bar, 100 μm.

To examine the cell line susceptibility to ZIKV Natal RGN SRIPs in vitro, the three cell lines TE-671, SF-268, and ARPE-19 were used (Figure 6). ZIKV Natal RGN SRIPs at a MOI of 0.15 TCID50/cell induced more observable and higher CPE levels in TE-671 cells than those in ARPE-19 and SF268 cells (Figure 6A top). The CPE level induced by ZIKV Natal RGN SRIPs correlated with the fluorescence intensity of the EGFP reporter encoded within the SRIP genome in the following order: TE-671 > ARPE-19 > SF268 cells infected with ZIKV Natal RGN SRIPs (Figure 6A middle). In addition, immunofluorescent staining with anti-ZIKV NS1 antibodies indicated that ZIKV NS1 protein was expressed in these three ZIKV Natal RGN SRIP-infected cell lines, in which the fluorescence intensity of the NS1 protein was consistent with the CPE level induced by ZIKV Natal RGN SRIPs (Figure 6A bottom). Meanwhile, 10^5^ TCID50 of the SRIPs per well was added onto the monolayer of these three cell lines to examine the attachment activity of ZIKV Natal RGN SRIPs (Figure 6B). Interestingly, the SRIPs showed the highest level of attachment activity to TE671 cells, in which the highest viral genome number was discovered 1 h after attachment at 4 °C. However, the SRIPs showed the lowest level of attachment activity to SF268 cells. Compared to ARPE-19 and SF268 cells, it was found that TE671 cells exhibited the highest susceptibility to ZIKV Natal RGN SRIPs and allowed the highest number of SRIPs to attach to the surface.

## 4. Discussion

The ZIKV Natal RGN strain found in brain tissues from fetal autopsy cases with microcephaly in the Natal region of Brazil has not been isolated [18]. This study, firstly, generated SRIPs of the ZIKV Natal RGN strain using synthetic and reverse genetic technologies (Figure 1, Figure 2, Figure 3 and Figure 4 and Supplementary Figures S1–S3). The viral yield of ZIKV Natal RGN SRIPs in prM-E-expressing packaging cells transfected with the ZIKV replicon was 6.25 × 10^6^ TCID50/mL (Figure 4), which indicated the replication capacity of the CMV promoter-launched ZIKV Natal RGN replicon. In addition, a green fluorescence reporter, EGFP, encoded within the ZIKV Natal RGN replicon, represented an observable marker to examine replication of the ZIKV Natal RGN replicon and SRIPs (Figure 2, Figure 3, and Figure 5). In our laboratory, the pBR322 plasmid has been used to construct a JEV replicon that is genetically stable in *E. coli* [15]. The JEV genome sequence encoding for structural proteins (C, prM/M, and E) was toxic in *E. coli*, which resulted in genetic instability of the JEV infectious clones and replicons [15]. Similarly, construction of ZIKV Natal RGN infectious clones was not accomplished due to the genetic instability of ZIKV Natal RGN infectious clones, particularly within the NS1 gene region (Supplementary Figures S1 and S2). However, the recombinant pcDNA3.1 plasmid containing prM and E genes and the pBR322 plasmid containing 5’ and 3’-UTRs, C, and all NS genes, showed genetic stability. Two ZIKV infectious clones obtained by cloning the entire full-length native cDNA have been reported [20,21], and other ZIKV infectious clones were reported using strategies to avoid the toxicity of the ZIKV genome sequence, including in vitro assembly of viral cDNA fragments and insertion of eukaryotic introns [22,23,24,25,26,27]. Particularly, the ZIKV Natal RGN infectious clone was produced using bacterial artificial chromosomes (BACs) [27], in which a single-copy pBeloBAC11 plasmid containing the full-length genome of the ZIKV Natal RGN strain under the CMV promoter control was used to maintain ZIKV genome stability and reduce toxicity in bacteria. In addition, our prior report demonstrated that JEV SRIPs carrying the EGFP reporter were a more sensitive, effective, and efficient platform for quantitating antiviral efficacy using flow cytometry compared to the traditional plaque assay [15]. Like JEV SRIPs, the ZIKV Natal RGN SRIPs carrying the EGFP reporter could be applicable as a drug screening platform. Therefore, the reverse genetics system for the production of ZIKV Natal RGN SRIPs provided an alternative, applicable, and rapid approach to studying the biological features of the unique ZIKV strains.

The human rhabdomyosarcoma/muscle TE671 cells were used as packaging cells expressing ZIKV prM and E proteins (Figure 2), in which transfection with the ZIKV Natal RGN replicon significantly generated recombinant SRIPs with the high yield of 6.25 × 10^6^ TCID50/mL (Figure 4). Interestingly, TE671 cells expressing JEV C, prM, and E proteins were also used as a packaging cell line that was efficient at producing JEV SRIPs post-transfection with the JEV replicon [15]. The recombinant ZIKV Natal RGN SRIPs showed infectivity and self-replication in the packaging cells (Figure 5). Two amino acid substitutions within NS5 (L3277R and E3280N) (Supplementary Figure S3) seemed to have no influence on the activities of NS5 methyltransferase and RNA-dependent RNA polymerase. A recent study indicated that human embryonic kidney 293T cells had been used to establish different flavivirus prM-E-expressing packaging cells, including DENV1–4, ZIKV, JEV, West Nile virus, yellow fever virus, and tick-borne encephalitis virus, and the cells were transfected with the DENV1 reporter replicon to generate a chimeric flavivirus SRIP-based neutralization assay [28]. Therefore, recombinant ZIKV Natal RGN SRIPs generated in this study could be used to analyze the ZIKV life cycle, elucidate viral pathogenesis, screen antiviral agents, design faster and safer diagnostic tools, and even develop attenuated vaccines against ZIKV infection.

The full-length ZIKV Natal RGN strain genome was directly sequenced using next-generation sequencing with the total RNA extracted from brain tissues from fetal autopsy cases with microcephaly (GenBank accession number KU527068) [18]. In this study, recombinant ZIKV Natal RGN SRIPs were rescued using reverse genetic and synthetic biology techniques (Figure 1, Figure 2, Figure 3, Figure 4 and Figure 5 and Supplementary Figures S1–S3). Three cell lines, TE671, SF268, and ARPE-19, were further tested for cell susceptibility to ZIKV Natal RGN *SRIPs* (Figure 6). The order of cell susceptibility to ZIKV Natal RGN SRIPs was TE671 > ARPE-19 > SF268 cells. The CPE level, EGFP reporter intensity, and ZIKV NS1 protein expression were most obvious (>50% involvement) in TE671 cells, but were mild in ARPE-19 cells and limited in SF268 cells 72 h post-infection with SRIPs at an MOI of 0.15 TCID50/cell. The results indicated that ZIKV Natal RGN *SRIPs* had a broad range of human tissue tropism with differential cell susceptibilities (Figure 6A). In addition, ZIKV Natal RGN SRIPs had the highest attachment activity to TE671 cells among those three cell lines (Figure 6B). SRIP attachment to TE671 cells was suggested to be involved in the infectivity of ZIKV Natal RGN in TE671 cells. This finding implied that ZIKV Natal RGN SRIPs had tropism for rhabdomyosarcoma/muscle and retinal pigmented epithelial cells, which was consistent with myalgia, arthralgia, retinitis, chorioretinal atrophy, and pigmentary maculopathy in symptomatic ZIKV infection [29,30]. Therefore, the cell line susceptibility results revealed the unique features of ZIKV Natal RGN SRIPs and suggested the possible pathogenesis of ZIKV infection.

## 5. Conclusions

In conclusion, the approach for generating recombinant ZIKV *SRIPs* with an EGFP reporter showed the genetic stability of ZIKV *prM*-*E* genes in the mammalian expression vector and ZIKV replicon sequence in the low-copy plasmid pBR322, providing a faster and safer platform to study the biological aspects of ZIKV infection. ZIKV Natal RGN SRIPs were rescued by the combination of prM-E-expressing packaging cells and the ZIKV Natal RGN replicon under CMV promoter control, which mimicked self-replicating, positive-sense, single strand RNA viruses. The biological features of ZIKV Natal RGN SRIPs were revealed, including CPE, infectivity, cell susceptibility, and attachment activity. Therefore, recombinant ZIKV Natal RGN SRIPs could be further used to investigate cell tropism, persistent infection, and vaccine candidates, and to establish reliable, effective, and efficient assays for screening antiviral agents and diagnosing viral infections.

## Supplementary Materials

The following are available online at https://www.mdpi.com/1999-4915/11/11/1005/s1, Supplemental Table S1. The primer pairs for cloning Zika virus replicon and prM-E gene into the expression vector; Supplemental Table S2. Primers for sequencing Zika virus replicon; Supplemental Table S3. Primer pairs for real-time quantitative RT-PCR of ZIKV E and NS5 RNA copies; Supplemental Figure S1. The construction of in-frame gene fusion (A), plasmid information and restriction enzyme digestion analysis (B) of the synthesized DNA segment I; Supplemental Figure S2. The construction of in-frame gene fusion (A), plasmid information and restriction enzyme digestion analysis (B) of the synthesized DNA segment II; Supplemental Figure S3. Sequencing analysis of ZIKV^Asian^_Natal RGN_ replicon with the primers listed in Supplemental Table S2A. The amino acid substitutions were also shown in the Figure.
