# Supplementary material for: The Rescue and Characterization of Recombinant, Microcephaly-Associated Zika Viruses as Single-Round Infectious Particles
Source: Viruses. 2019 Oct 31;11(11):1005. doi: 10.3390/v11111005 (PMC6893733; doi:10.3390/v11111005)
Supplement: Supplementary file 1 [file viruses-11-01005-s001.pdf]

Supplemental Table 1. The primer pairs for cloning Zika virus replicon and prM-E gene into the expression vector

Primer	Primer sequence (5’→3’)	Restriction enzyme site
A For EcoRI-CMV	5'- GATTACGAATTCTTGACATTGATTATTG -3'	EcoRI
A Rev 2A-NotI	5'- GTATGTACGCGGCCGCGGGCCCGGGGTTGGACTCAACGTC -3'	NotI
B1F NotI-E-NS1	5'- GAAGCTCGCGGCCGCATTGGGCAAGGGCATCCATC -3'	NotI
B1R NS1-Link1	5'- CTCTTGTTCCACATGTTTCCTCCACG -3'	
B2F NS1-Link2	5'- GAATGCCCGGGCACTAAGGTCCACGTGG -3'	
B2R NS-ClaI-XhoI	5'- GCAGCTCGAGTTCAGCATCGATGGCTCGAAGCACTC -3'	ClaI、XhoI
CF ClaI-NS	5'- GAGCCATCGATGCTGAAGAAGAAGCAGCTAACTGTC -3'	ClaI
CR NS-RsrII-XhoI	5’- TTCTCGAGAGGCGGTCCGCGACAGTATGG -3’	RsrII、XhoI
DF NS-RsrII	5’- CCATACTGTCGCGGACCGCCT -3’	RsrII
DR BGH-XhoI	5'- GACGACCTCGAGCCATAGAGCCCACCGCATCCCCAGC -3'	XhoI
prM/E-For	5'- AACTTAAGCTTACCATGCGTGGGAGTGCATACTATATGTACTTGGACAGAAAC -3'	EcoRI
prM/E-Rev	5'- TCTAGACTCGAGTTACACATCAGCAGAGACGGCTG -3'	XhoI

Supplemental Table 2. Primers for sequencing Zika virus replicon

**Primer**	**Primer sequence (5'→3')**
CMV seq-63-80F	5'-CATATATGGAGTTCCGCG-3'
ZIKV seq-305-324	5'-CAATAGATGGGGTTCAGTGG-3'
ZIKV seq-1902-1920	5'-ACCGCAGCGTTCACATTCA-3'
ZIKV seq-2855-2873	5'-CTTCGTCAGAGCAGCAAAG-3'
ZIKV seq-3701-3718	5'- TGACCTGGCTAAGCTTGC -3'
ZIKV seq-4661-4680	5'- GGAGACCACAGATGGAGTGT -3'
ZIKV seq-5502-5520	5'- GCAAGAGGATACATTTCAA -3'
ZIKV seq-6460-6478	5'- AAAGAGGAGCGGCTTTTGG -3'
ZIKV seq-7301-7319	5'- AGCAGCTGCGCGTGCTGCC -3'
ZIKV seq-8257-8275	5'- GACTGCAGCGTAGGTATGG -3'
ZIKV seq-9102-9120	5'- TGGCTAGGGGCTAGATTTC -3'
ZIKV seq-10051-10069	5'- CTACCTGGTCAATCCATGG -3'
P3 Rev (SacI)	5'- AGCCTGAGCTCCAGGCTCTC -3'
Zika C1-Rev	5'- ACACAGTGAAGTGGCTG -3'
Zika-NS5 Rev(2)	5'- GTGGTGGGAGCAAAACGGAACTT -3'

Supplemental Table 3. Primer pairs for real-time quantitative RT-PCR of ZIKV E and NS5 RNA copies

Primer	Primer sequence (5’→3’)
**ZIKV-E For**	5’- CAAGATCCCGGCTGAAACACTG- 3'
**ZIKV-E Rev**	5’- TTCTCCCCGACTCCTATGACAATG- 3’
**ZIKV-NS5 For (antisense cDNA synthesis)**	5’- ACCCTGGGATGTGGTG -3’
**ZIKV-NS5 Rev (sense cDNA synthesis)**	5’- GTTGAGGGTTTCCACTCTTG -3'
**Zikz-NS5 For(2)**	5’- CTTGTGGCTGCTGCGGAGGTCA -3'
**Zikz-NS5 Rev(2)**	5’- GTGGTGGGAGCAAAACGGAACTT -3'

A.

TTGACATTGATTATTGAC………{CMV promoter nt1-601}…. GCTGGTTTAGTGAACCGT
agttgttgatctgtgtga……..{ZIKV^{Asian} Natal RGN strain nt1-500}…actagacgtgggagtgca
ATGGTGAGCAAGGGCGAG……{EGFP nt1-717}……….………ATGGACGAGCTGTACAAG
AACTTTGATTTGCTCAAG……..{FMDV-2A nt1-51}……...……GAGTCCAACCCCGGGCCC
tactatatgtacttggac…{ZIKV^{Asian} Natal RGN strain nt501-3364}…..cgtggaggaaacatgtgg

B.

Plasmid No.	160506SV7732-3MR30	Storage	-20° C
Cloning Site	EcoRI/Acc65I	Host	STBL3
Resistance		Plasmid Qty.	4μg/Vial
Note	BBI keep the stock of plasmid and a copy of sequencing report for 3 months.		

Restriction Enzyme Digestion Analysis:

Results	Description
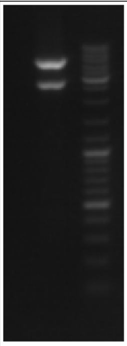	Enzyme: EcoRI/Acc65I<br>Expected Size: 4733+2.7k<br>Marker Ladder: 100bp, 200bp, 300bp, 400bp, 500bp, 600bp, 700bp, 800bp, 900bp, 1000bp, 1200bp, 1500bp, 2000bp, 2500bp, 3000bp, 3500bp, 4000bp, 5000bp, 6000bp, 8000bp, 10000bp

Supplemental Figure 1. The construction of in-frame gene fusion (A), plasmid information and restriction enzyme digestion analysis (B) of the synthesized DNA segment I.

A.

cgggcactaaggtccacg..{ ZIKV^{Asian} Natal RGN strain nt3331-10808}.gggaaatccatgggtctt

GGGTCGCCCAGGAATGGC….{HDVr nt1-79}………..……..GATGGCCGGCATGGTCCC

CGACTGTGCCTTCTAGTT…...{bGH PA terminator nt1-228}… ATGCGGTGGGCTCTATGG

B.

Plasmid No.	160603V7294-2MR4	Storage	-20° C
Cloning Site	EcoRI/Acc65I	Host	top10
Resistance		Plasmid Qty.	4μg/Vial
Note	BBI keep the stock of plasmid and a copy of sequencing report for 3 months.		

Restriction Enzyme Digestion Analysis:

Results	Description
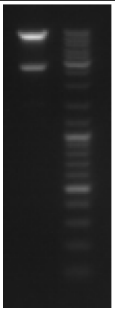	Enzyme: EcoRI/Acc65I<br>Expected Size: 7784+2. 7k<br>Marker Ladder: 100bp, 200bp, 300bp, 400bp, 500bp, 600bp, 700bp, 800bp, 900bp, 1000bp, 1200bp, 1500bp, 2000bp, 2500bp, 3000bp, 3500bp, 4000bp, 5000bp, 6000bp, 8000bp, 10000bp

Supplemental Figure 2. The construction of in-frame gene fusion (A), plasmid information and restriction enzyme digestion analysis (B) of the synthesized DNA segment II.

A.

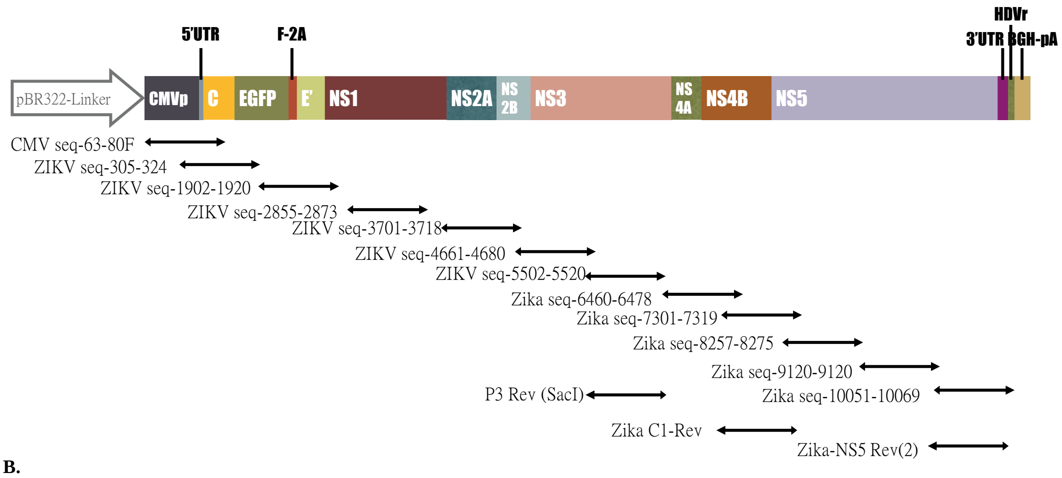

B.

No.	Protein	Codon mutation	Amino acid substitution
1	NS1	ACA → ACC	T829T
2	NS3	GTT → GTA	V2962V
3	NS5	CTG → CGG	L3277R
4	NS5	GAA → AAC	E3280N

Supplemental Figure 3. Sequencing analysis of $\text{ZIKV}^{\text{Asian}}{}_{\textit{Natal}}$ RGN replicon with the primers listed in Supplemental Table 2 (A). The amino acid substitutions were also shown in the Figure.
